# Metabolic engineering for high yield synthesis of astaxanthin in *Xanthophyllomyces dendrorhous*

**DOI:** 10.1186/s12934-021-01664-6

**Published:** 2021-09-06

**Authors:** Alejandro Torres-Haro, Jorge Verdín, Manuel R. Kirchmayr, Melchor Arellano-Plaza

**Affiliations:** Biotecnología Industrial, Centro de Investigación y Asistencia en Tecnología y Diseño del Estado de Jalisco, A.C. Camino Arenero 1227, Col. El Bajío del Arenal, 45019 Zapopan, Jalisco Mexico

**Keywords:** Astaxanthin, Carotenoids, Biological pigments, Metabolic engineering, Omics-based engineering, *Xanthophyllomyces dendrorhous*

## Abstract

Astaxanthin is a carotenoid with a number of assets useful for the food, cosmetic and pharmaceutical industries. Nowadays, it is mainly produced by chemical synthesis. However, the process leads to an enantiomeric mixture where the biologically assimilable forms (3R, 3′R or 3S, 3′S) are a minority. Microbial production of (3R, 3′R) astaxanthin by *Xanthophyllomyces dendrorhous* is an appealing alternative due to its fast growth rate and easy large-scale production. In order to increase *X. dendrorhous* astaxanthin yields, random mutant strains able to produce from 6 to 10 mg/g dry mass have been generated; nevertheless, they often are unstable. On the other hand, site-directed mutant strains have also been obtained, but they increase only the yield of non-astaxanthin carotenoids. In this review, we insightfully analyze the metabolic carbon flow converging in astaxanthin biosynthesis and, by integrating the biological features of *X. dendrorhous* with available metabolic, genomic, transcriptomic, and proteomic data, as well as the knowledge gained with random and site-directed mutants that lead to increased carotenoids yield, we propose new metabolic engineering targets to increase astaxanthin biosynthesis.

## Introduction

Carotenoids have gained biotechnological importance due to their benefits to human health. They are powerful antioxidative and anti-inflammatory agents, as well as efficient UV radiation protectants. In addition, they are precursors of retinol (Vitamin A) and other derivatives that participate in cell regeneration. Therefore, carotenoids are interesting for food, cosmetic and pharmaceutical industries [1–5]. Among the carotenoids with the highest biotechnological potential are phytoene, lycopene, β-carotene, zeaxanthin and astaxanthin [1, 6, 7].

Astaxanthin (3,3′-dihydroxy-β, β′-carotene-4,4′-dione) is probably one of the most interesting and valuable carotenoids. Its antioxidant activity has been reported to be 10 and 100 times higher than those of β-carotene and α-tocopherol, respectively [8–10]. It is estimated that by the end of 2020 there will be a turnover in carotenoids sales of around $1.85 billion USD. The sales of astaxanthin represents 29% of the total with a 2.3% annual increase [11]. Total astaxanthin sales by 2025 are estimated to be $2.57 billion [12]. Synthetic astaxanthin price is around 2500 USD/kg, but the price increases to about $100,000 USD/kg for natural and high purity astaxanthin [13–15].

**Fig. 1:**
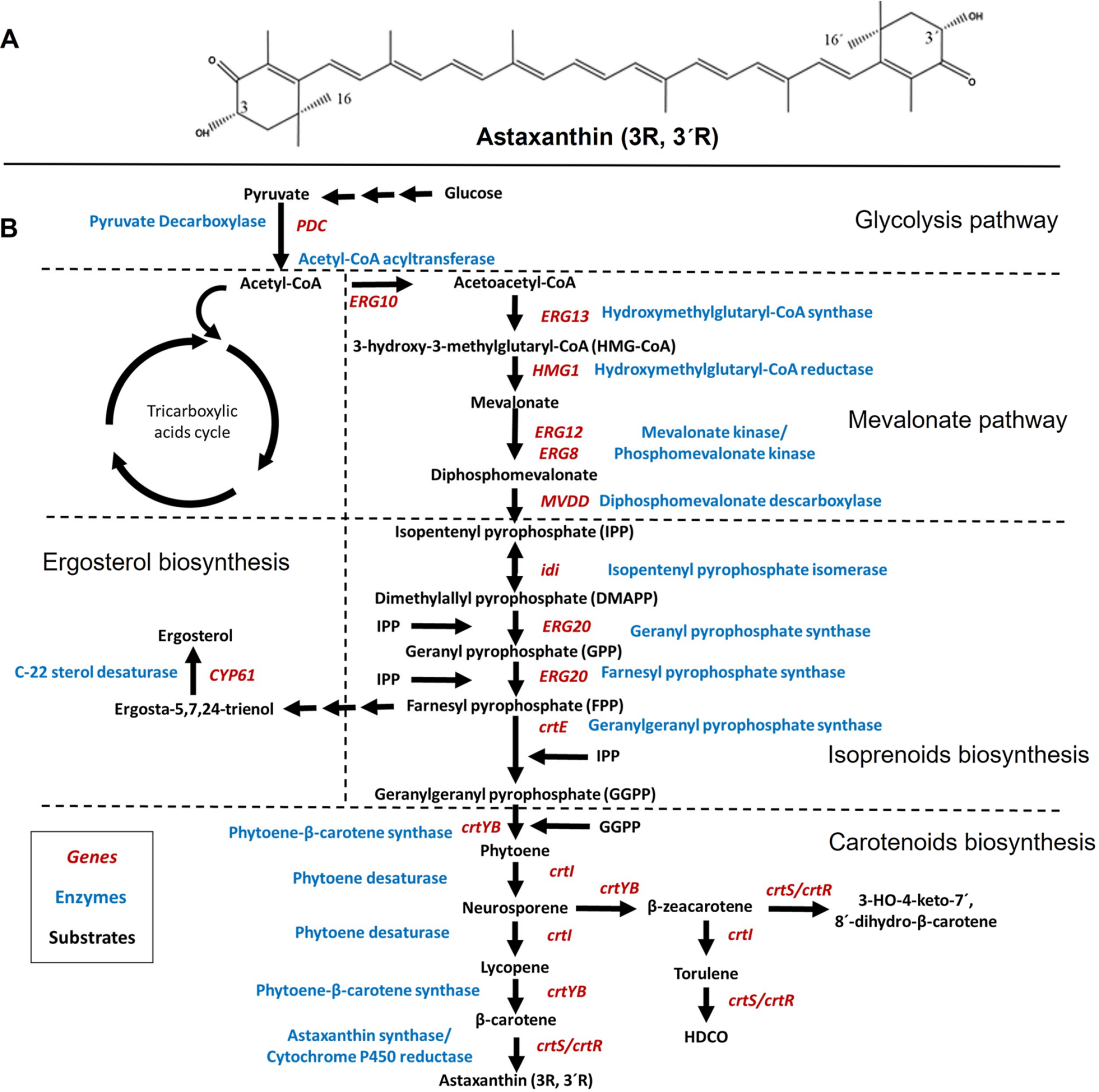
**A** Chemical structure of the astaxanthin (3R, 3R) isomer produced by *X. dendrorhous*. **B** General scheme of astaxanthin biosynthesis pathway in *X. dendrorhous*. Genes, enzymes, and substrates that participate in the metabolic pathway are shown in red, blue, and black, respectively

Value differences between natural and synthetic astaxanthin arise from the composition of the enantiomeric mixture each source produces, which is also related to their biological activity. There are three possible astaxanthin stereoisomers: (3R, 3′R), (3R, 3′S) and (3S, 3′S). Natural astaxanthin is predominantly (3S, 3′S) or (3R, 3′R), while synthetic astaxanthin contains a mixture of all three possible enantiomeric forms, (3R, 3′R), (3R, 3′S), and (3S, 3′S), in a 1:2:1 ratio, respectively [16–18]. Because of the enantiomeric mixture, synthetic astaxanthin shows relatively low biological activity [17–19]. On the other hand, the enantiomeric form of natural astaxanthin may vary from one organism to another; (3R, 3′R) is the main astaxanthin stereoisomer produced by *Xanthophyllomyces dendrorhous* (Fig. 1A) [17, 20], while the (3S, 3′S) form is mainly produced by *Haematococcus pluvialis* [17, 21].

Bernhart et al. [16] analyzed the natural accumulation of different astaxanthin stereoisomers in *Meganyctiphanes norvegica*, and two different species of salmonids: Atlantic salmon (*Salmo salar*) and Pacific salmon (*Oncorhynchus tshawytscha*). They observed that (3S, 3′S)-astaxanthin accumulated more (77–85%) than (3R, 3′R) stereoisomers (11–18%). However, Foos et al. [17] reported a similar absorption of both (3R, 3′R) and (3S, 3′S) stereoisomers (90% and 96%, respectively) in rainbow (*Oncorhynchus mykiss*) and sea (*Salmo trutta*) trout. Similarly, a study in humans showed that the absorption of both (3R, 3′R and 3S, 3′S) stereoisomers was also similar [19].

In terms of bioactivity, non-esterified (3S, 3′S and 3R, 3′R)-astaxanthin applied to lymphocytes and peritoneal exudates of mice [22], promote cell proliferation, killer cell cytotoxic and phagocytic activity. Nevertheless, non-esterified (3S, 3′S)-stereoisomer showed a slightly higher immunoregulatory effect than the other one. (3R, 3′R)-astaxanthin shows specific activities as lipoperoxidation reducing agent in rainbow trout [23], antioxidant capable of delaying aging [24] and, in chickens, increases immunoglobulin and splenocyte proliferation [25].

*Xanthophyllomyces dendrorhous* natively produces around 97% of non-esterified (3R, 3′R)-astaxanthin [14, 26]. In contrast, *H. pluvialis* produces (3S, 3′S)-astaxanthin in high yields, but it is esterified by palmitic, stearic or linoleic acids [13, 14, 17, 18], which requires hydrolysis by lipases before it can be assimilated. The assimilation of astaxanthin esters may be variable due to the complexity of the molecule and the organism assimilation capacity [16, 17, 19]. Storebakken et al. [18] reported a lower absorption of astaxanthin dipalmitate esters (47%) than free astaxanthin (64%) in Atlantic salmon.

Nowadays, most astaxanthin is produced by chemical synthesis; nevertheless, microbial sources are well known. As can be inferred from previous paragraphs, the main producers of this pigment are the yeast *X. dendrorhous* and the microalgae *H. pluvialis* [1, 12, 26–30]. Nevertheless, microbial production of astaxanthin is less competitive than the synthetic one because of difficulties to obtain enough biomass and its low pigment yield, thus overproducing astaxanthin from appropriate microbial sources is a current biotechnological challenge [1, 12, 31]. So far, bioengineering approaches that include culture media and bioreactor optimization [30], as well as construction of improved overproducing strains [12, 31, 32], have been assayed. Metabolic engineering of *Saccharomyces cerevisiae*, *Candida utilis*, *Pichia pastoris* and *X. dendrorhous* have led to increased amounts of phytoene, lycopene or β-carotene, but they have failed to increase astaxanthin biosynthesis in relevant levels [1, 30, 33–35].

Here, we envisage new metabolic engineering targets to generate *X. dendrorhous* strains able to shift the carbon metabolic flux towards astaxanthin biosynthesis. To that end, random and site-directed genetic modifications made to *X. dendrorhous* and other yeasts were analyzed from their consequences in gene expression to the impact in astaxanthin yield.

## *Xanthophyllomyces dendrorhous* biology

The yeast *X. dendrorhous* (also known as *Phaffia rhodozyma* in its teleomorphic state) is a basidiomycete that exceptionally produces xanthophylls in response to lipoperoxidation caused by environmental stress [36, 37]. In addition to pigments, *X. dendrorhous* genome encodes a vast battery of anti-oxidative enzymes such as catalases and superoxide dismutases [38]. *X. dendrorhous* life cycle is homothallic and characterized by the mating of a mother cell with a bud (paedogamy) on polyol-rich media. After mating, a non-septated basidium (holobasidium) is formed with mononuclear basidiospores arising on its apex [37, 38]. These basidiospores lead to diploid vegetative cells [39–41], although haploid strains have been characterized [42]. Unlike basidiomycetous yeasts, *X. dendrorhous* does not show a transition from unicellular to filamentous growth during the sexual phase.

The elucidation of the genome sequence of several *X. dendrorhous* strains (19.5 megabases, and 6385 protein coding genes in CBS 6938 strain) showed an atypical high rate of introns per gene (7.4–7.5) [38, 42]. Additional genomic analysis also revealed the existence of two main acetyl-CoA derived biosynthetic pathways, terpenoid and fatty acid biosynthesis [42]. Terpenoids in *X. dendrorhous*, like in any other fungus, are synthesized via the mevalonate pathway and typically ends with ergosterol synthesis. However, as shown in the next sections, the mevalonate pathway in *X. dendrorhous* bifurcates, leading to the carotenoids and sterols pathways. Unlike other carotenogenic fungi, genes involved in sterols and carotenoids biosynthesis in *X. dendrorhous* are not organized in clusters.

## Biosynthesis of astaxanthin in *X. dendrorhous*

Astaxanthin derives from the isoprene biosynthesis pathway (Fig. 1B). Acetyl-CoA produced after glycolysis is introduced into the mevalonate pathway to yield isopentenyl pyrophosphate (IPP) [17, 20]. IPP can be isomerized in dimethylallyl pyrophosphate (DMAPP) by bifunctional IPP isomerase (*idi*). Later, geranyl pyrophosphate (GPP) and farnesyl pyrophosphate (FPP) are synthesized by the action of FPP synthase (*ERG20*). Afterwards, the ligation of IPP with FPP, mediated by GGPP synthase (*crtE*), leads to geranylgeranyl pyrophosphate (GGPP). The condensation of two GGPP molecules carried out by phytoene-β-carotene synthase (*crtYB*) leads to phytoene. Subsequently, four dehydrogenations on phytoene are performed by phytoene desaturase (*crtI*) to synthesize lycopene. Two cyclization steps catalyzed by phytoene-β-carotene synthase (*crtYB*) are needed to finally produce γ-carotene and β-carotene. Astaxanthin formation involves the oxidation of β-carotene by astaxanthin synthase (*crtS*) and cytochrome P450 reductase (*crtR*) [43–47]. After astaxanthin biosynthesis, lipoproteins transport the molecule into biological membranes for its storage and cell protection [6].

Glyceraldehyde 3-phosphate and pyruvate also contribute to the mevalonate pathway since the metabolic flux of these compounds produce acetyl-CoA [48–50]. Nevertheless, pyruvate and acetyl-CoA are also amino acid and fatty acids precursors, as well as substrates of the tricarboxylic acids cycle (Fig. 1B) [51]. Although these substrates cannot be exclusively dedicated to the astaxanthin biosynthesis, it is possible to stress the yeast, either modulating the fermentation conditions or adding precursors and cofactors, to improve astaxanthin yields [30, 49].

*Xanthophyllomyces dendrorhous*, despite its high capacity to accumulate astaxanthin, shows a number of limitations that hampers its synthesis [26, 52]. Within the mevalonate pathway, hydroxymethylglutaryl-CoA reductase (*HMG1*), the main supplier of the pathway (Fig. 1B), is regulated by product and, therefore, it is a limiting step that hinders the carbon flux [53]. In addition, the accumulation of 3-hydroxy-3-methylglutaryl-CoA, substrate of the aforesaid reductase, is toxic to cells, which explains the low expression levels of genes involved in its synthesis. A second limiting step is the reaction catalyzed by mevalonate kinase (*ERG12*) whose expression is regulated by isoprenoid products, mainly geranyl pyrophosphate and farnesyl pyrophosphate [54].

Within the astaxanthin biosynthesis pathway, phytoene-β-carotene synthase (*crtYB*) is also a bottleneck for the accumulation of astaxanthin due to its low expression levels [33, 55–58]. Similarly, low expression levels of cytochrome P450 reductase (*crtR*), which participates in the catalysis of the last step of the astaxanthin biosynthesis, have been observed [51, 56, 59–61]. Moreover, the biosynthesis of carotenoids is inactivated when catabolite repression is induced. In *X. dendrorhous*, Mig1 has been identified as a catabolic repressor of carotenoids biosynthesis [62].

Table 1 shows the enzymes and encoding genes directly involved in the astaxanthin biosynthesis, and their subcellular localization within *X. dendrorhous.*

**Table 1:** Genes/enzymes involved in the biosynthesis of astaxanthin by *X. dendrorhous*

Gene name	Gene ID	Enzyme name	Subcellular localization^a^	References
*ERG10*	CED82454	Acetyl-CoA acyltransferase	Cytoplasm, MIS	[42, 126, 129, 130]
*ERG13*	CED83016	Hydroxymethylglutaryl-CoA synthase	Nucleus	[42, 126, 129, 130]
*HMG1*	CED85502	Hydroxymethylglutaryl-CoA reductase	Nucleus periphery, ERM	[42, 126, 129, 130]
*ERG12*	CED82307	Mevalonate kinase	Cytoplasm	[42, 126, 129, 130]
*ERG8*	CDZ97430	Phosphomevalonate kinase	Cytosol and nucleus	[42, 126, 129, 130]
*MVDD*	CED83492	Diphosphomevalonate decarboxylase	Cytosol	[42, 126, 129, 130]
*idi*	CED82414	Isopentenyl pyrophosphate isomerase	Cytoplasm and nucleus	[42, 126, 129, 130]
*ERG20*	CDZ96456	Farnesyl pyrophosphate synthase	ERM	[42, 126, 129]
*crtE*	CDZ97186	Geranylgeranyl pyrophosphate synthase		[42, 126, 129, 130]
*crtYB*	CED83449	Phytoene-β-carotene synthase		[126, 129, 130]
*crtI*	CED83513	Phytoene desaturase	ICM	[126, 129, 130]
*crtS*	CED83940	Astaxanthin synthase		[126, 129, 130]
*crtR*	CDZ98161	Cytochrome P450 reductase	MOM, ERM, PM	[126, 129, 130]

*MIS* mitochondrial intermembrane space, *ERM* endoplasmic reticulum membrane, *BM* vacuole membrane, *ICM* integral component of membrane, *MOM* mitochondrial outer membrane, *PM* plasma membrane

^a^Reference [129]

## Biotechnological production of astaxanthin by *X. dendrorhous*

*Xanthophyllomyces dendrorhous* is the most widely used microorganism in industry for astaxanthin production since, using appropriate substrates, it is its main carotenoid (85% of total, approximately) [26]. However, the low yield of astaxanthin produced by the yeast (which varies between 200 and 400 µg/g of dry biomass) and the high costs of culture media, have hampered its use as a large-scale source [20, 30, 46, 63]. Moreover, the chemical synthesis of astaxanthin has a final yield of 8% in relation to supplies employed in the process [64]. This value is higher than the biological production of astaxanthin in *X. dendrorhous* whose yield lays between 0.02 and 0.03% with respect to the substrate employed [1, 63, 65, 66].

In order to make microbial production more competitive, it is obviously necessary to increase the yield [12, 30, 31, 63]. Nutrimental and operational fermentation conditions have been optimized to stimulate astaxanthin biosynthesis in *X. dendrorhous*, among which can be accounted carbon source concentration (15.0 to 35.0 g/L), nitrogen (0.5 to 3.0 g/L), pH (5.5 to 6.0), temperature (18.0 to 22.0 °C), dissolved oxygen (above 20%) [29, 30, 65, 67–71], white light (177 μmol photon/m^2^ × s) [13, 72], and inoculum size (5–10%) [69]. Dissolved oxygen has been elucidated as one of the most important factors in *X. dendrorhous* fermentation. Wu et al. [73], by means of a transcriptional analysis, showed that supply of 25% dissolved oxygen leads to 2.5-fold increase of *crtYB* and *crtI* expression, and 1.5-fold increase of *crtR* and *crtS* expression. Regarding nutritional requirements, glucose and ammonium sulfate are the preferred carbon (C) and nitrogen (N) sources, which maximize astaxanthin production when they are employed in a high C/N molar ratio (usually between 40 and 76) [45, 46, 51]. In addition, glutamate and yeast/malt extracts increase the assimilation of C/N for astaxanthin biosynthesis [74].

Other strategies to increase astaxanthin yields in *X. dendrorhous* and other yeasts, even more promising than fermentation optimization, is the generation of random [75, 76] or site-directed [30, 33, 55, 77] overproducing mutant strains [8, 30, 47, 49, 75, 79].

## Physical chemical mutagenesis of *X. dendrorhous* to increase astaxanthin biosynthesis

Random mutagenesis has been set up for the isolation of carotenoids-overproducing *X. dendrorhous* strains. These strategies consist in the exposure of yeast cells to physical and chemical mutagenic agents [8, 78–80] such as ultraviolet and gamma radiation, 1-methyl-3-nitro-1-nitrosoguanidine (NTG) [8, 45, 79–81] and/or ethyl methane sulfonate (EMS) [29, 45]. Because these strategies lead to a large number of mutant colonies, it is necessary to implement efficient selection methods to identify the best mutants [68, 79]. Astaxanthin and/or sterol biosynthesis inhibitors in the culture medium such as β-ionone, diphenylamine, ketoconazole, miconazole, 2-methylimidazole, clotrimazole, nicotine, nystatin, mevinolin, pyridine, and *N*,*N*-diethylamine facilitate the selection of mutants that overproduce carotenoids above wild-type levels because the mutant-enhanced metabolic pathway (carotenoids or biosynthetic precursors) can overcome the effect caused by the inhibitor [78, 79].

Gui-Li et al. [4] obtained *X. dendrorhous* Y119, a strain able to produce 6.4 mg astaxanthin/g of dry biomass, by exposing yeast cells to EMS. Jeong-Hwan et al. [8] and Ang et al. [78] reported the use of NTG to generate JH1 and M34 strains, respectively. JH1 achieved an astaxanthin production above 4 mg/g of dry biomass, while M34 only yielded 0.5 mg/g, i.e., 2 to 50 times more astaxanthin than wild-type strains in batch culture. These results illustrate the great variability of the output of random mutagenesis approaches [17, 72].

In order to obtain genetically stable *X. dendrorhous* strains, Chun et al. [82] performed a recombination method in which, in the first instance, native strains were subjected to chemical mutagenesis with NTG. The resulting strains produced carotenoids above 1.6 mg/g of dry biomass after induction with antimycin A [46, 79] which, due to its chemical properties, alters the electron transport chain and, consequently, the mitochondria. These physiological affectations activate the cellular defense machinery, i.e., the biosynthesis of carotenoids [79]. After selection of the best mutants, they were genetically backcrossed by each other’s protoplast fusion to eliminate deleterious mutations. The resulting hybrids were mostly stable and capable of hyperproducing carotenoids in yields greater than 2 mg/g of dry biomass.

Random mutagenesis may have negative effects on cell physiology, viability, growth and metabolic capacity that cannot be overlooked [45, 79, 83]. These mutants are unstable and frequently lose their overproduction ability because of active cell repairing mechanisms [79, 84, 85]. Alternative strategies to develop stable mutants is the knocking-out and/or overexpression of genes directly or indirectly involved in the astaxanthin biosynthesis using site-specific molecular tools [30, 75, 86].

## Metabolic engineering of *X. dendrorhous*

The increased availability of metabolic engineering tools has had a major impact on the development of *X. dendrorhous* strains that overproduce astaxanthin [12, 30–32, 49, 86–88]. These strains have been designed under the principle of maximizing the carbon flow from the basic substrates towards the astaxanthin biosynthesis route, minimizing the generation of unwanted byproducts (Fig. 1B) [31, 49, 51, 88]. Shifting the carbon flux towards astaxanthin biosynthesis has been achieved by spotting key enzymes and bottlenecks of the metabolic pathway [31, 33, 51, 74], optimizing the expression levels of relevant genes, and thereby increasing the storage of astaxanthin in cells [33, 49, 51, 74, 86].

**Fig. 2:**
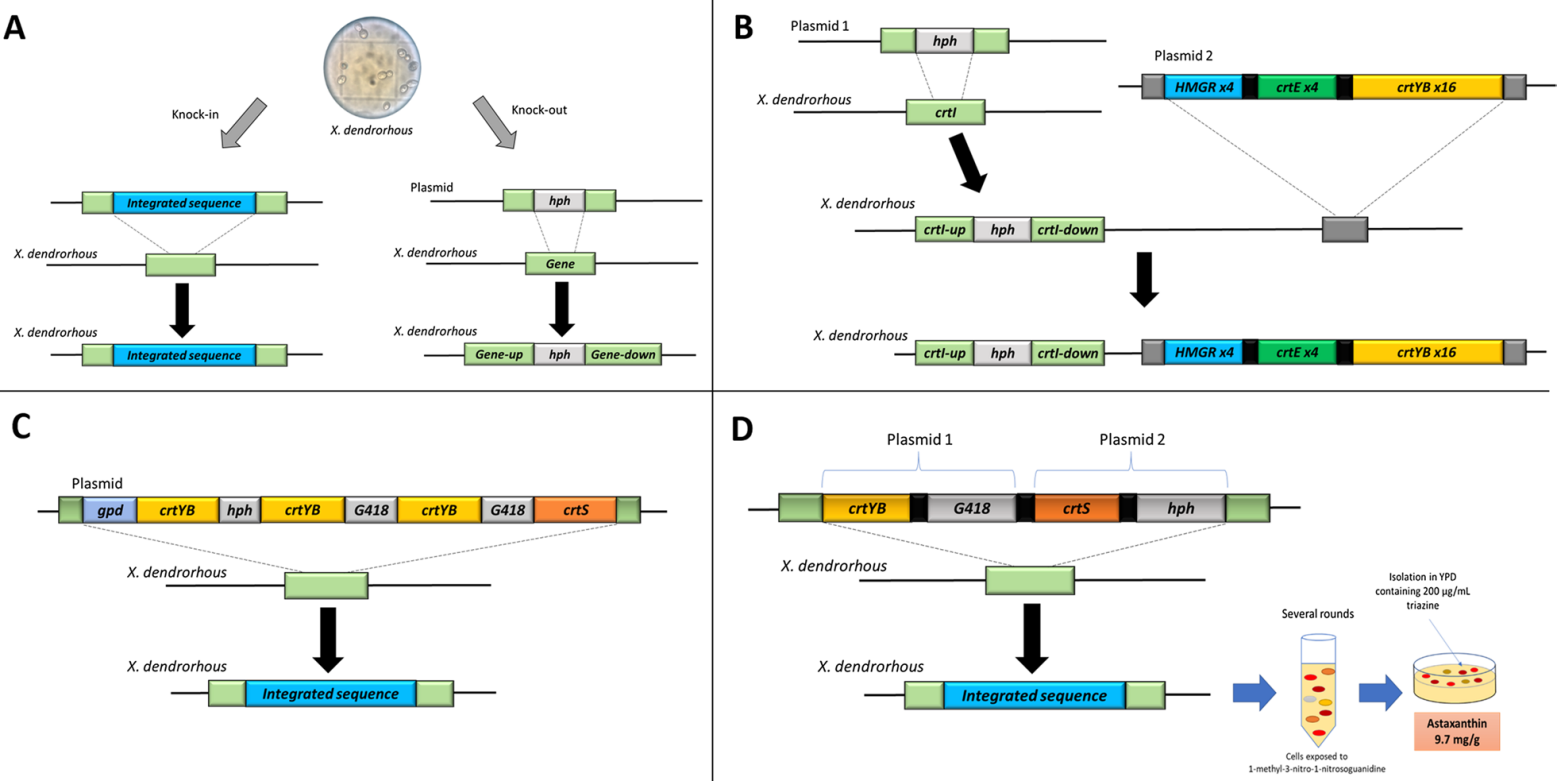
Metabolic engineering of *X. dendrorhous* for overproduction of carotenoids. **A** Representation of knock-in and knock-out strategies for genetic engineering in yeast. **B** Overexpression of *HMG1*, *crtE* and *crtYB* for high production of phytoene [58]. **C** Genetic engineering of *X. dendrorhous* for high yield astaxanthin production. Integration of multiple copies of *crtYB* and *crtS* to increase the production of β-carotene and its conversion to astaxanthin, respectively [33, 89]. **D** Metabolic engineering and conventional mutagenesis for high yield astaxanthin production [66]. *crtYB* and *crtS* genes were integrated using geneticin (G418) and hygromycin (*hph*) as selection markers. Afterwards, a random chemical mutagenesis was carried out using 200 µg/mL NTG. Chemical mutagenesis was repeated several rounds until the maximal astaxanthin overproducer was obtained. The best strain obtained, AXJ-20/*crtYB*, was able to yield above 9.7 mg/g of dry biomass

Besides the availability of *X. dendrorhous* genome sequence [42], efficient integrative vectors that contain positive selection markers such as *nptII* (G418) or *hph* (hygromycin), have been developed (Fig. 2). Those vectors have been used to knock-out or overexpress genes under the control of strong constitutive promoters. Recently, genetic engineering strategies have been implemented in *X. dendrorhous* to increase isoprenoids accumulation and consequent carotenoids biosynthesis [12, 30–32, 49, 86–88]. Moreover, Cre/*lox-P* systems have been used to remove selection markers after integration in *X. dendrorhous* genome [59]. Those molecular tools, in addition to the characteristic genetic tractability of *X. dendrorhous*, have stimulated an abundant metabolic engineering literature [12, 30, 31, 49].

In the next sections, intervention of the *crt* gene family that encodes enzymes of the carotenoids biosynthesis pathway is discussed.

### Overexpression/inactivation of the *crtYB* gene

One of the main targets of metabolic engineering has been *crtYB*, a gene that encodes for phytoene-β-carotene synthase (Table 1), the bifunctional enzyme with phytoene cyclase and β-carotene synthase activities responsible for the phytoene and β-carotene synthesis [54, 56, 58, 89].

Phytoene is the first carotenoid of the astaxanthin biosynthesis pathway (Fig. 1B). This carotenoid has a growing market as a skin protector and food supplement [90]. *X. dendrorhous* is a potential natural source of this compound (Table 2, Fig. 1B). Pollmann et al. [58] introduced genetic modifications to *X. dendrorhous* to obtain high yields of phytoene. To achieve this goal, *crtI* was knocked down to block the carbon flow and allow phytoene accumulation (Fig. 1B). The phytoene biosynthesis was further improved by integrating 4 copies of *HMG1* (encoding hydroxymethylglutaryl-CoA reductase). In addition, 16 copies of *crtYB* and 4 copies of *crtE* were introduced (Fig. 2B). The resulting strain was able to produce 7.5 mg phytoene/g biomass. In a continuous culture at a small scale, a greater efficiency was achieved with phytoene yields of more than 10 mg/g biomass. This result is higher than in any other reported organism [58], which demonstrates the potential of *X. dendrorhous* for carotenoids biosynthesis [30].

**Table 2:** Strains of *X. dendrorhous* and *S. cerevisiae* genetically engineered to overproduce carotenoids/astaxanthin

Microorganism	Genetic modification scheme	Mutagenesis method	Targeted gene	Product	Yield (mg/g)	References
*X. dendrorhous*	None			Astaxanthin	0.2 to 0.4	[14, 44, 63]
	Random	EMS	Random	Astaxanthin	6.4	[4]
		NTG	Random	Astaxanthin	4	[79, 80]
		NTG	Random	Astaxanthin	0.5	[78]
		NTG		Astaxanthin	2	[82]
	Site-specific	Gene duplication	*crtYB*	β-Carotene	0.65	[89]
		Gene duplication	*crtYB*	Astaxanthin	0.49	[56]
		Inactivation	*CYP61*	Astaxanthin	0.28	[88]
		Gene duplication	*crtE*,* crtS*	β-Carotene	0.47	[59]
		Inactivation *crtI*/gene duplication	*HMG1*, *crtE*, *crtYB*	Phytoene	10	[58]
		Inactivation *crtS*/gene duplication	*crtZ*, *crtYB*	Zeaxanthin	5.2	[61]
		Gene duplication/NTG	*crtYB*,* crtS*	Astaxanthin	9.7	[66]
*S. cerevisiae*	Site-specific	Heterologous integration	*crtYB*, *crtE*, *crtI*	Lycopene	23	[92]
		Heterologous integration	*crtE*, *crtYB*, *crtI*	β-Carotene	6.01	[100]
		Heterologous integration/protein engineering	*crtE*,* crtI*, *crtYB*, *crtZ*, *bkt*	Astaxanthin	8.1	[96]

Exploring the consequences of knocking *crtYB* out with a *nptII*-containing integrative cassette, Visser et al. [33] isolated white colonies unable to synthesize carotenoids, which demonstrated the importance of *crtYB*. This result confirmed the work of Verdoes et al. [55], who found that *crtYB* is a limiting step in the biosynthesis of carotenoids and astaxanthin [43–45, 47, 49].

Realizing the importance of phytoene-β-carotene synthase (*crtYB*), and keeping active the basal *crtI* function, multiple copies of *crtYB* were introduced into PR-1-104, a *X. dendrorhous* random mutant that already overproduced β-carotene (1 mg/g dry biomass) [89]. Overexpression of phytoene-β-carotene synthase in such genetic background led to 1.5 times higher β-carotene accumulation than the parental strain.

In an independent work, Ledetzky et al. [56] integrated 2 and 3 copies of *crtYB* in different strains and, in addition to an increase of canonical carotenoids, they detected the biosynthesis of a new carotenoid never described before, 3-HO-4-keto-7′,8′-dihydro-β-carotene. Both strains were able to produce more than 0.7 mg carotenoids/g of dry biomass, of which 40% was astaxanthin. The biosynthesis of new carotenoids was rationalized by the fact that phytoene synthase/lycopene cyclase can perform cyclizations in carotenoids. This enzyme has been shown to take phytoene and neurosporene as substrates to catalyze the sequential biosynthesis of β-zeacarotene, 7,8-dihydro-β-carotene and, with the participation of *crtR* and *crtS* [43, 44, 47, 75], 3-HO-4-keto-7′,8′-dihydro-β-carotene (Fig. 2B) [56]. *crtS* encodes for astaxanthin synthase that belongs to the cytochrome P450 family [75, 86], which has monooxygenase activity (catalyzes the β-carotene hydroxylation and ketolation). However, *crtS* depends on *crtR* since the latter, due to its reductase activity, provides the necessary electrons to astaxanthin synthase to catalyze the substrate oxygenation [75, 86].

*crtYB* overexpression is essential to increase carotenoids biosynthesis, but to overproduce astaxanthin it is necessary to overexpress astaxanthin synthase (*crtS*) [75]. Because of this, Ledetzky et al. [56] generated a *X. dendrorhous* strain containing three copies of *crtYB* and one of *crtS*, whose astaxanthin biosynthesis increased to 70% of total carotenoids produced (Fig. 2C) [57].

Nevertheless, the described increase of carotenoids synthesis is not enough to compete against the production costs of chemical synthesis. Therefore, it is necessary to explore other genes/enzymes of the metabolic pathway involved in astaxanthin biosynthesis.

### Overexpression of the *crtI* gene

The gene *crtI* encodes phytoene desaturase in *X. dendrorhous*, the second enzyme of the carotenoid biosynthesis pathway (Fig. 1B). The introduction of several copies of *crtI* increased the accumulation of intracellular lycopene [89, 91], which generated pink to dark red colonies of the yeast. Despite an increase of β-carotene and astaxanthin was expected, their concentration in fact decreased. Nevertheless, an increase of torulene and 3-hydroxy-3,4-didehydro-β, Ψ-carotene-4-ona (HDCO; Fig. 1B) was detected [89, 91]. This implies that the over expression of the phytoene desaturase in *X. dendrorhous* shifts the metabolic flux towards torulene biosynthesis (Fig. 1B) [80, 89].

Xie et al. [92], in order to overproduce lycopene, constructed a *S. cerevisiae* strain heterologously expressing in vitro evolved copies of *crtYB*, *crtE* and *crtI* genes from *X. dendrorhous*. They first inactivated *crtYB* in *X. dendrorhous* and, after applying directed evolution schemes to the inactivated *crtYB*, as well as the active *crtE* and *crtI*, they obtained mutant strains with phytoene synthase activity able to accumulate phytoene, but decreased accumulation of β-carotene. Evolved *crtYB*, *crtE*, and *crtI* were introduced into *S. cerevisiae* to generate a strain that produced more than 23 mg lycopene/g of dry biomass. Lycopene accumulation was possible since *S. cerevisiae* does not express astaxanthin synthase (*crtS*), responsible for the astaxanthin biosynthesis, HDCO and other unwanted byproducts of the astaxanthin biosynthesis pathway (Fig. 1B).

Similarly, Yamada et al. [93] constructed a *S. cerevisiae* strain (YTPH499/Mo3Crt79) that produced 6.01 mg β-carotene/g dry biomass in 96 h. The transformation was achieved through the simultaneous integration of three copies of *crtE*, one copy of *crtYB* and one copy of *crtI*. All genes were isolated from *X. dendrorhous*.

In accordance with the discussed results, it is necessary to find metabolic engineering alternatives that do not divert the carbon flow towards the synthesis of torulene, HDCO or unwanted byproducts but that allow the accumulation of astaxanthin as the main product.

### Overexpression of the *crtS* gene

Astaxanthin synthase, encoded by *crtS* in *X. dendrorhous*, together with cytochrome P450 reductase (*crtR*), catalyzes the last step of the astaxanthin pathway transforming β-carotene into astaxanthin. This is the limiting step in the astaxanthin biosynthesis. As previously mentioned in “Overexpression/inactivation of the crtYB gene” section, *crtS* has monooxygenase activity (catalyzes the β-carotene hydroxylation and ketolation) that depends on *crtR* activity [75, 86].

Gassel et al. [66] carried out the integration of additional copies of *crtYB* and *crtS* in *X. dendrorhous* genome to increase astaxanthin production (Fig. 2D). Afterwards, random chemical mutagenesis with NTG (200 µg/mL) was performed. Surviving colonies were exposed to the same treatment several rounds until the maximum astaxanthin producer was isolated, AXJ-20/*crtYB.* This strain was able to produce > 9.7 mg astaxanthin/g of dry biomass, after isolating it in 200 µg/mL triazine-containing media. Chemical mutagenesis could alter other genes involved in astaxanthin biosynthesis [66]. For this reason, it is necessary to determine the transcriptomic and proteomic profiles of obtained strains.

**Fig. 3:**
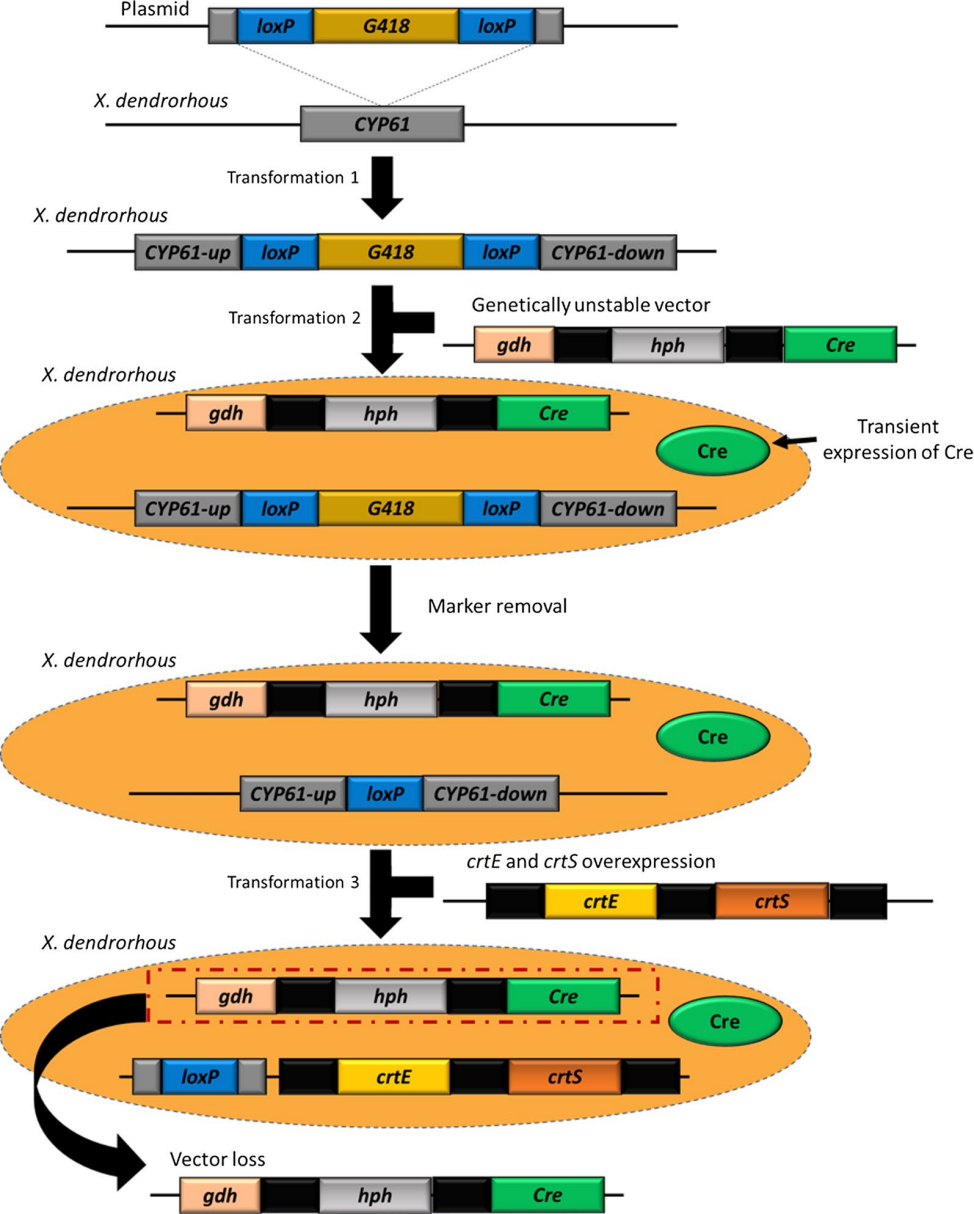
Integration of additional copies of *crtE* and *crtS* using a Cre/*lox-P* system [59]. The Cre–*loxP* recombination system allows selectable markers recycling*.* Holding the transitory expression of *cre* (encoding Cre recombinase) controlled by an unstable vector, the selectable marker flanked by *loxP* sites were removed along with the Cre vector

Contreras et al. [75] generated two new *X. dendrorhous* strains that contained either one (Xs_1H1S) or two (Xd_2H2S) copies of *crtS*. While the control strain, UCD 67–385, achieved astaxanthin yields of 128 µg/g dry biomass, Xs_1H1S and Xd_2H2S reached a production of 154 µg/g and 179 µg/g biomass in 96 h, respectively. *X. dendrorhous* has been also transformed with additional copies of *crtS* and *crtE* using a Cre–*lox-P* system (Fig. 3). This strain, Xd-ES, was able to increase β-carotene and astaxanthin yields above 0.47 mg/g and 0.55 mg/g of dry biomass, respectively [59]. Nevertheless, the yield obtained is not yet enough to compete with astaxanthin produced by chemical synthesis or from improved microbial sources obtained by random mutagenesis.

Since *crtS* acts together with *crtR* [75, 86] to generate astaxanthin, it is necessary to explore the overexpression of both genes [49, 51].

### Importance of *crtR* in the carotenoids metabolic pathway

*crtR* encodes cytochrome P450 reductase, which is involved in the last limiting step of the astaxanthin biosynthesis pathway in *X. dendrorhous* [61]. As was mentioned in “Overexpression/inactivation of the crtYB gene” section, *crtR* provides electrons to astaxanthin synthase, which catalyzes β-carotene oxygenation leading to astaxanthin synthesis [75, 86]. In order to explore the relationship of *crtR* with *crtS* and its importance in the biosynthesis of the compound, Alcaíno et al. [86] inactivated *crtR* in two wild-type strains of *X. dendrorhous*, CBSTr and T13. Both strains were pale because they were unable to synthesize astaxanthin or only in minimal amounts, but accumulated β-carotene in concentrations of 114 and 50 mg/L, respectively. Parental strains were able to produce only 9 mg/L β-carotene and above 100 mg/L astaxanthin. These results revealed the importance of *crtR*, whose knock down led to accumulation of β-carotene and concomitantly stopped the astaxanthin production [86]. Furthermore, the deletion generated in both wild-type strains affected differently as indicated by the β-carotene production obtained. These results also show that, even among wild *X. dendrorhous* strains, there may be a high level of polymorphism since the same genetic modification affected differently the metabolic capacity of each engineered strain [40, 86].

Some authors have stated that it is improbable to obtain *X. dendrorhous* overproducing mutants due to low *crtR* expression levels. It is necessary to integrally study the regulation of *crtR* and *crtS*, which work together in the conversion of β-carotene to astaxanthin, since they have only been studied separately [86]. Additionally, there are thirteen cytochrome P450 genes [94] whose products are monooxygenases that could be involved in both primary and secondary metabolism (such as sterol biosynthesis, carotenoid biosynthesis and aromatic compound degradation). Alcaíno et al. [94] characterized the genes that encode other two monooxygenases belonging to cytochrome P450 family, which participate in the sterol biosynthesis: *CYP51* and *CYP61* (encoding for lanosterol 14-alpha demethylase and C-22 sterol desaturase, respectively). Since *CYP51* and *CYP61* are also involved in ergosterol biosynthesis, they have the ability to decrease the flux of available carbon for astaxanthin production (Fig. 1B).

Yamamoto et al. [88] reported a complete *CYP61* deletion in a *X. dendrorhous* diploid strain. The result was a promising strategy to decrease ergosterol biosynthesis and, consequently, increase the carbon flux towards astaxanthin biosynthesis (Fig. 1B), obtaining 1.4-times higher astaxanthin production compared to the parental strain. This metabolic engineering strategy is based in the fact that ergosterol, a product of lipid biosynthesis, is an inhibitor of *ERG13* and *HMG1*, which act in the synthesis of mevalonate (Fig. 1B). In addition, the *CYP61* disruption in wild-type *X. dendrorhous* strains increased the *HMG1* expression levels from 2 to 5 times during astaxanthin biosynthesis [95]. It is necessary to insightfully study cytochrome P450 monooxygenases since some other genes could be related directly or indirectly to the astaxanthin metabolic pathway [57, 79, 88, 94, 95].

### Astaxanthin biosynthesis in heterologous hosts

**Fig. 4:**
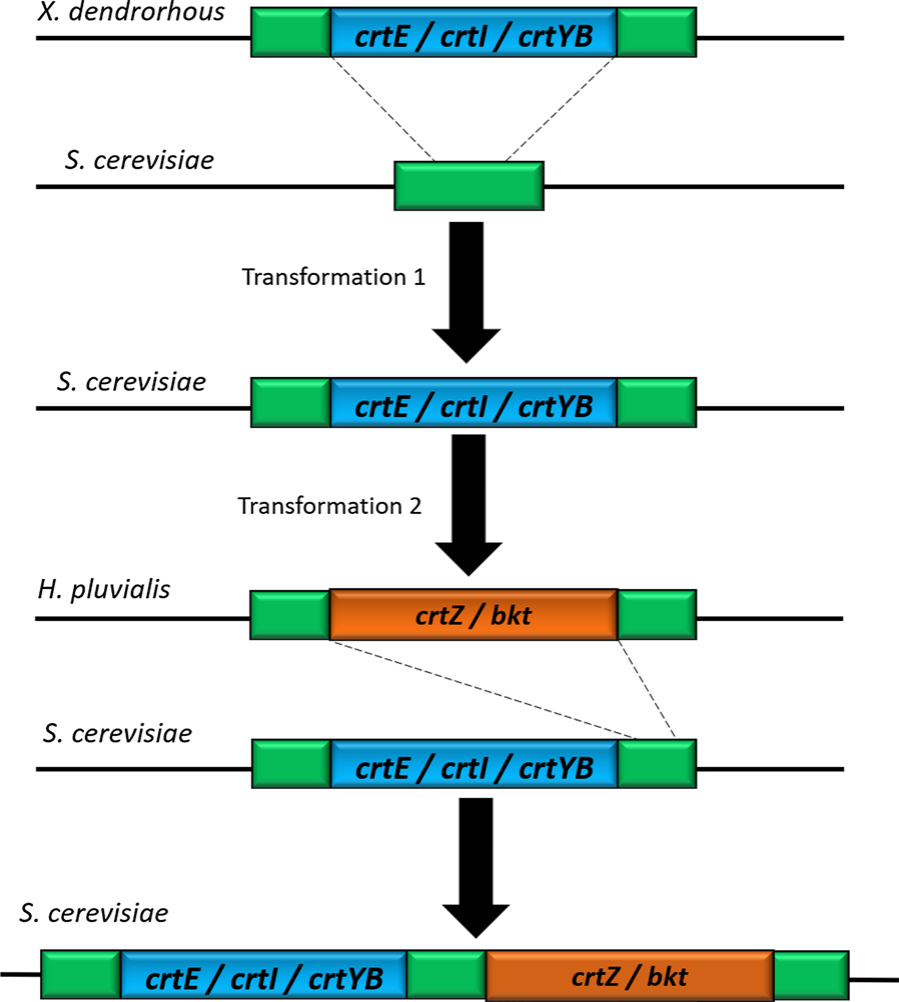
Metabolic engineering in *S. cerevisiae* for astaxanthin (3S, 3′S) overproduction [96]. A β-carotene overproducer *S. cerevisiae* strain was obtained by heterologously expressing *crtE*, *crtI* and *crtYB* genes from *X. dendrorhous*. Subsequently, *crtZ* and *bkt* from the microalgae *H. pluvialis* were expressed. *crtZ* encodes for β-carotene hydroxylase which catalyzes the conversion of canthaxanthin to astaxanthin. *bkt* encodes for β-carotene ketolase which catalyzes the conversion of β-carotene to canthaxanthin

Astaxanthin biosynthesis has been recreated in *S. cerevisiae* by integrating heterologous genes encoding the necessary enzymatic machinery. Zhou et al. [96] generated a *S. cerevisiae* strain (YastD-01) capable of producing astaxanthin in high yields (up to 8.1 mg/g dry biomass). As a first step, they built a β-carotene producing strain by heterologously expressing *crtE*, *crtI* and *crtYB* from *X. dendrorhous*. Subsequently, they expressed *crtZ* and *bkt* from *H. pluvialis*. *crtZ* encodes β-carotene hydroxylase, which catalyzes the conversion of canthaxanthin to astaxanthin. *bkt* encodes β-carotene ketolase, which catalyzes the conversion of β-carotene to canthaxanthin [96]. The insertion of both *H. pluvialis* genes led to a yield of 8.1 mg astaxanthin/g of biomass, but it turned out to be the (3S, 3′S) stereoisomer. The graphic representation of this genetic engineering scheme is shown in Fig. 4. Similarly, Jiang et al. [97] generated a recombinant *S. cerevisiae* strain by introducing the *carRA* (orthologous to *crtYB* from *X. dendrorhous*) and *crtI* genes from *Blakeslea trispora*, *crtZ* from *Agrobacterium aurantiacum*, *crtW* from *Brevundimonas vesicularis*, which encodes a β-carotene ketolase (non-encoded in *X. dendrorhous* genome) and *crtE* from *Taxus media*. The transforming *S. cerevisiae* strain was only capable of producing 20 mg astaxanthin/L, but, additionally, it was subjected to several rounds of physical mutagenesis and adaptation to hydrogen peroxide. After 15 rounds, the best selected strain was able to produce > 65 mg astaxanthin/L in a batch system, while in a fed batch system the maximum concentration obtained was 404.78 mg/L.

In terms of (3R, 3′R)-astaxanthin overproduction, it has not yet been achieved in satisfactory levels. Table 2 shows a summary of the most relevant works on metabolic engineering for astaxanthin biosynthesis, both in *X. dendrorhous* and *S. cerevisiae*. The highest astaxanthin yield to date is around 10 mg/g biomass. Since *X. dendrorhous* is the microorganism that possesses the native enzymatic machinery for the astaxanthin biosynthesis, it could be the option with the greatest potential for metabolic engineering. However, it is still necessary to study in greater detail the carbon flux and the metabolic limitations that exist in this biological system, the goal we are pursuing in this review.

## Metabolic strategies to increase the carbon flux to astaxanthin biosynthesis *in X. dendrorhous*

Nowadays, metabolic engineering of *X. dendrorhous* has not led to competitive astaxanthin (3R, 3′R) overproducing mutants. In addition to the use of mevalonate, citrate, glutamate, succinate, glycerol and other molecules as inductors of enzymes that participate in the astaxanthin biosynthesis [30, 74, 98, 99], it is necessary to establish the influence of sugars metabolism and the tricarboxylic acids cycle in the carbon flow towards the carotenoids biosynthesis [48, 49]. This information can be used to identify enzymes that participate in those metabolic pathways that increase the flux towards astaxanthin accumulation.

In species such as *Candida utilis*, *S. cerevisiae* and other yeasts, a strong limitation in the expression levels of HMG-CoA reductase and GGPP synthase has been found and, subsequently, are limited the levels of mevalonate and isoprenoids derivatives available for carotenoid and astaxanthin biosynthesis [30, 31, 35, 49, 100]. Analogously, this could be one of the reasons why the carbon flux in *X. dendrorhous* could be reduced for isoprenoids, lipids and carotenoid biosynthesis (Fig. 1B).

In *X. dendrorhous*, phytoene-β-carotene synthase is a key factor to displace the metabolic flux to astaxanthin biosynthesis from its substrate, GGPP [30, 31, 33]. If there is not enough substrate for astaxanthin biosynthesis, the carbon flux shifts to isoprenoids and sterols biosynthesis (Fig. 1B) [30, 31, 42]. It has been demonstrated that the inactivation of the gene that encodes for squalene synthase in *C. utilis* and, in parallel, overexpression of HMG-CoA reductase, increases the carotenoid biosynthesis up to 2 times [30, 31, 35]. Similarly in *S. cerevisiae*, decreasing the regulation of *ERG20* by inactivation of squalene synthase and increasing GGPP synthesis by overexpression of *crtE*, causes an increase in carotenoids synthesis [31]. Moreover, as mentioned earlier in “Importance of crtR in the carotenoids metabolic pathway” section, Yamamoto et al. [88] demonstrated that the complete deletion of *CYP61* (C-22 sterol desaturase) in *X. dendrorhous* increases *HMG1* expression levels from 2 to 5 times [95] and, subsequently, increases the carbon flux towards astaxanthin biosynthesis (production above 1.4 times). This is due to the decrease of ergosterol biosynthesis, which is an inhibitor of *ERG13* and *HMG1* activities (Fig. 1B). In addition, *CYP61* deletion in *X. dendrorhous* leads to an increased *SRE1* expression level (around 3.5 times compared with wild-type strains) [101] with which the metabolic pathways derived from terpenoid biosynthesis are activated, and thus astaxanthin and ergosterol precursors production.

Nevertheless, high yields of carotenoids may affect the cell since their intracellular accumulation could be toxic [49, 52]. However, increasing the ability of the host to accumulate big amounts of lipids is possible [102] using directed evolution techniques [32] or overexpressing genes that encode enzymes involved in the cell membrane flexibility. In *S. cerevisiae*, it has been observed that overexpression of stearoyl-CoA 9-desaturase increases the capacity to accumulate lycopene up to 30% [103], while overexpressing *ino2*, which encodes a transcriptional factor involved in the response to physiological stress, increases up to 10% the ability to accumulate lipids and derivatives (including lycopene) [104].

To determine which are the metabolic mechanisms that influence the carotenoids and astaxanthin biosynthesis in *X. dendrorhous*, proteomic and transcriptomic analysis have been performed on overproducing strains [76, 81]. Barbachano-Torres et al. [76], using random mutagenesis with NTG on a wild-type strain, obtained an XR4 mutant able to synthesize 2.5–8.8 times more astaxanthin than the parental strain. The proteomic profile of the mutant was 90% identical to the wild-type strain; however, they found a decrease in NADP(H)-dependent glutamate dehydrogenase 1 (GDH1) and UDP-glucuronic acid decarboxylase; an increase of NADP(H)-dependent glutamate dehydrogenase 3 (GDH3), glyceraldehyde 3-phosphate dehydrogenase (GPDH), dihydrolipoyl dehydrogenase and a probable pyridoxine synthase. GDH1 and GDH3 are involved in ammonium assimilation and glutamate biosynthesis, respectively [43, 105, 106]. Glutamate has recently been shown to aid in carbon and nitrogen assimilation, which increases astaxanthin biosynthesis [106, 107]. On the other hand, dihydrolipoyl dehydrogenase participates in the metabolism of tricarboxylic acids, which is why it promotes the generation of reactive oxygen species and the subsequent biosynthesis of carotenoids in response to oxidative damage [76, 108]. GPDH is related to the production of high-energy molecules during glycolysis, and it is likely that other oxidation reactions are triggered including those that lead to cellular apoptosis. In response to this oxidative damage, genes involved in astaxanthin biosynthesis are transcribed [44, 51]. UDP-glucuronic acid decarboxylase is related to the metabolism and assimilation of sugars, which is why it maintains the cellular integrity [76, 109]. It is probable that the overexpression of these enzymes could play an important role in the generation of overproducing strains. A transcriptomic analysis of XR4 mutant strain (capable of overproducing 2.5–8.8 times more astaxanthin than the wild-type strain) [76, 81] revealed that it overexpresses *crtE* and *crtS*, and *crtI* at levels 3 and 2 times above the control strain, respectively. However, expression levels of genes such as *idi* were variable, while *crtYB* and *crtR* expression was even lower than those of the control strain. According to the expression levels of the genes involved in the carotenoids and astaxanthin biosynthesis, *crtS* action is essential to astaxanthin biosynthesis while *crtR* could be expressed at non-limiting levels in this overproducer *X. dendrorhous* strain. Nevertheless, the *crtR* expression levels may be variable and limiting between different mutant strains due to their polymorphism [40, 86] and different capacities to displace carbon flux towards the different metabolic pathways [84].

Pan et al. [106] demonstrated that using a 76:1 C/N ratio is critical to increase the concentration of astaxanthin in *X. dendrorhous* UV3-721 mutant strain. After a proteomic analysis, they found 1.5- to 2.5-fold more Grg2, aspartic protease precursor, transaldolase, 3-isopropylmalate dehydrogenase and a disulfide isomerase precursor compared to that observed at low 19:1 C/N ratio. They also observed sixfold overexpression of phospho-methyl-pyridine kinase, 3.3 times more astaxanthin synthase, and overexpression of IPP isomerase and pyruvate decarboxylase. On the other hand, in this study, unlike the results reported by Barbachano et al. [76], a tenfold decrease in GPDH expression was observed. This may be due to the high C/N ratio used as a metabolic stressor to induce the generation of carotenoids instead of high energy molecules in the tricarboxylic acid cycle. In addition, overexpression of other key enzymes such as pyruvate decarboxylase, IPP isomerase and astaxanthin synthase, strongly correlated with the biosynthesis of carotenoid precursors and astaxanthin [51, 59].

The strategy of feeding 0.368 g/L glutamate in media culture, preserving a high 76:1 C/N ratio, has been used [74]. Glutamate participates in the tricarboxylic acids cycle in the form of 2-ketoglutarate due to the activity of GDH. Because of that, it is an activator of carbon and nitrogen assimilation, which provides energy to the cell and leads to a 40% increase in astaxanthin biosynthesis [74, 76]. Under these conditions, a proteomic analysis showed a 2–2.5-fold overexpression of phytoene desaturase, GDH, glycerate and formate dehydrogenase, IPP dehydrogenase and cytochrome P450 reductase. There was also a 1.3–1.5-fold overexpression of dihydrokaempferol 4-reductase, 3-isopropyl malate dehydrogenase and astaxanthin synthase. These data were corroborated by assessing the corresponding expression levels. All these enzymes play a key role in the primary and secondary metabolism, and evidently influence the astaxanthin biosynthesis. This is why it is important to consider them in the metabolic engineering processes to generate astaxanthin overproducing strains [74].

Proteomic analysis has revealed that the oxidative stress generated by the tricarboxylic acid cycle is a key factor in increasing the expression levels of genes involved in astaxanthin biosynthesis, as well as of precursor compounds [51, 74, 76, 106]. The use of succinate (20 g/L) as a carbon source has been used by Wozniak et al. [98] to induce astaxanthin biosynthesis, obtaining 2.5 times more astaxanthin compared to that obtained when glucose (20 g/L) is used as carbon source. Through a transcriptional analysis, it was shown that succinate, after the exponential growth phase, increased the expression levels of *crtYB* (4.2- to 4.5-fold), *crtI* (1.7- to 1.8-fold) and *crtS* (up to 1.3-fold) compared to the use of glucose as carbon source [98].

Moreover, decreasing the expression levels of Mig1 in *X. dendrorhous* leads to twofold increase of *crtYB* and *crtS* expression levels [62]. However, it was observed that the expression of *crtI* in the mutant strain was lower than that of the wild-type one (− 2 times). Since *crtS* and *crtR* work simultaneously in the astaxanthin biosynthesis (Fig. 1B), it has been reported that *crtS* overexpression and maintaining non-limiting *crtR* expression levels [76, 86], resulted in 5.5-fold increase of astaxanthin biosynthesis.

Mig1 is a catabolic repressor that binds to specific DNA sequences called “*Mig1* boxes” in the promoter regions of various glucose-repressed genes in yeast [62, 98]. Mig1 union sites in *crtYB* and *crtI* have been identified, while two sites were detected on the regulatory region of *crtS* gene [98]. Mig1 is regulated by phosphorylation. In the absence of glucose, Mig1 is phosphorylated by Snf1 kinase, and allocated to the cytoplasm. However, in the presence of glucose, an intracellular signaling cascade activates the dephosphorylation of Mig1, which subsequently migrates towards the nucleus where it binds to the transcriptional corepressor complex [110, 111]. Therefore, decreasing the Mig1 expression levels represents an alternative to increase the carbon flow towards the synthesis of astaxanthin in *X. dendrorhous*.

Taking advantage of cell division acceleration induced by plant cytokinin 6-benzylaminopurine (6-BAP) [112], Pan et al. [51] managed to increase biomass (up to 3.33 g/L) and astaxanthin production (4.67 mg/g dry biomass). The metabolome of 6-BAP-treated yeast revealed glycolysis induction (although the rate of carbohydrate consumption decreased), and suppression of the tricarboxylic acids cycle that prevented diverting the carbon flow to unwanted metabolic routes. 6-BAP also triggered oxidative stress leading to increased trehalose biosynthesis (from glucose) and accumulation of astaxanthin in response to the high concentration of reactive oxygen species [51, 113, 114].

Transcriptional analysis of 6-BAP treated *X. dendrorhous* showed overexpression of astaxanthin biosynthesis genes [51]. *HMG1* and *idi* showed a significant 3.5-fold overexpression, while *crtE* had a twofold increase. *crtS* and *crtYB* had a 1.7 and twofold increase, respectively [51]. *crtI*, in the middle of the fermentation process, had a 1.35-fold expression increase, but at the end it decreased to its basal level [51]. It is important to note that it is necessary to maintain *crtI* expression levels close to basal since increasing its activity can divert the carbon flux towards the generation of torulene and HDCO [89, 91]. Regarding maximization of carbon flux towards mevalonate and carotenoids biosynthesis, modulation of all these genes by 6-BAP significantly increased the substrate availability for subsequent accumulation of astaxanthin [53].

The synthesis of HMGR and NADPH is also important in the mevalonate metabolic pathway for the synthesis of carotenoid precursors [49, 115–117]. Within the possible scenarios of metabolic engineering in the tricarboxylic acid cycle, overproduction of NADPH [49, 50, 118], through the overexpression of citrate synthase, succinate dehydrogenase and glutamate dehydrogenase, in addition to the overproduction of malate dehydrogenase, could increase the accumulation of carotenoid precursors and push the carbon flux towards astaxanthin biosynthesis in *X. dendrorhous*.

The *X. dendrorhous* Sre1 functions as a sterol regulatory element-binding protein (SREBP) [101, 119]. The transcription factor domain of Sre1 (the N-terminal domain) has a DNA binding motif [120] and the C-terminal domain (regulatory domain) interacts with a protein called SCAP (SREBP cleavage activating protein, named Scp1 in *S. cerevisiae*) that inhibits sterols biosynthesis when the cellular levels are sufficient. When sterol levels drop, the Sre1–Scp1 complex is transported to the Golgi apparatus, leading to Sre1 release, and translocation to the nucleus. Then, transcription of genes involved in sterol biosynthesis is activated [121], and concomitantly carotenoids biosynthesis decreases [86]. However, *SRE1* deletion inhibits both sterol and carotenoid biosynthesis [101, 119]. Gutierrez et al. [119] showed the N-terminal domain overexpression of *SRE1* increases 1.5 and twofold the expression of *ERG13* and *HMG1*. This could be used as an alternative to the isoprenoids overproduction to increase the astaxanthin biosynthesis.

In recombinant carotenoids-producing *S. cerevisiae*, mevalonate kinase (*ERG12*) and farnesyl pyrophosphate synthase (*ERG20*) have been recently identified as limiting factors for the isoprenoid accumulation in carotenoid biosynthesis [122]. Using CRISPR/Cas9 [32, 122], a *S. cerevisiae* strain able to accumulate up to 11 times more isoprenoids was obtained. *ERG12* and *ERG20*, in a similar way to *S. cerevisiae*, could generate a bottleneck for the astaxanthin biosynthesis in *X. dendrorhous*.

The genomic, transcriptomic and proteomic data described above, indicate it is necessary to increase the carbon flux towards astaxanthin biosynthesis. Knowing that the mevalonate pathway and its isoprenoid products feed carotenoid biosynthesis [17, 20, 49, 51], it is necessary to simultaneously overexpress *HMG1*, *ERG12*, *ERG20* and *idi* genes that encode enzymes that limit the accumulation of FPP, the main substrate for carotenoid biosynthesis. Despite the latter has been performed in *S. cerevisiae* and *Y. lipolytica*, this metabolic engineering program has not been carried out in *X. dendrorhous*. If increasing FPP accumulation is achieved, a second step focused on increasing the expression of genes directly involved in the astaxanthin biosynthesis pathway is necessary [33, 51, 55–59, 61]. The low activity of *crtYB*, *crtR* and *crtS* are the main bottlenecks in the synthesis of astaxanthin and their overexpression would also prevent the accumulation of isoprenoid products, thus avoiding the effect of inhibition by products such as sterols.

Among other important considerations, decrease of catabolic repression in *X*. *dendrorhous* activates the entire metabolic machinery for carotenoid/astaxanthin biosynthesis, a process that results from the inactivation of *MIG1* and, subsequently, increase of *crtYB* and *crtS* expression levels [62, 110, 111].

Overexpressing stearoyl-CoA 9-desaturase (*OLE1*) has been shown to increase the accumulation of substrates for carotenoid biosynthesis [103] or, in contrast, deletion of *CYP61* increases the substrates availability for astaxanthin biosynthesis and inhibition by sterols is evaded [88]. On the other hand, overexpressing enzymes involved in the tricarboxylic acid cycle such as NADPH oxidase, citrate synthase, succinate dehydrogenase and glutamate dehydrogenase may increase the carbon flux [49–51, 118] to astaxanthin accumulation.

The presence of oxygen, including reactive oxygen species such as H_2_O_2_, stimulates the production of carotenoids and astaxanthin as a protection mechanism in response to oxidative cell damage [69, 123–126]. Due to the above, it is proposed to turn off the enzymatic machinery in charge of evading reactive oxygen species so that carotenoids/astaxanthin biosynthesis be the main antioxidative response. Therefore, turning off *CTT1*, which encodes for catalase in *X. dendrorhous*, as well as knocking out the genes that encode for superoxide dismutase (*SOD1*) and glutathione peroxidase, may further induce astaxanthin biosynthesis. In *X. dendrorhous*, four copies of *CTT1* have been found [126]. In *S. cerevisiae*, it has been found a great diversity of glutathione peroxidases encoded by the genes *URE2*, *GPX1*, *GPX2*, *GTT1* and/or *hyr1p* [126] able to prevent intracellular oxidative damage.

Another potentially powerful strategy to favor secondary metabolism in *X. dendrorhous* and thereby promote the biosynthesis of carotenoids and astaxanthin is to keep the biological system under constant oxidative stress throughout the fermentation process. Taking advantage of the knowledge gained about the addition of H_2_O_2_ as an inducer of the production of astaxanthin [123], it is possible to obtain a genetically modified strain able to produce H_2_O_2_ autonomously. Within the genome of basidiomycota fungi, the superfamily of glucose-methanol-choline (GMC) oxidoreductases includes enzymes that produce extracellular H_2_O_2_ involved in the degradation of structural plant polysaccharides such as lignin, cellulose and hemicellulose [127, 128]. Within this superfamily can be found glucose oxidase (GOX), glucose dehydrogenase (*GDH*), ethanol/methanol oxidase (*MOX*), aryl-alcohol oxidase (*AAO*), pyranose 2-oxidase (*P2O*), pyranose dehydrogenase (*PDH*) and glyoxal oxidase (*GLX*). Heterologously expressing some of these genes in *X. dendrorhous* and, in addition, knocking out the reductases (catalase, superoxide dismutase and glutathione peroxidase) necessary to avoid oxidative damage, could favor the overproduction of astaxanthin.

## Conclusions

As discussed above, there are a number of different strategies to obtain genetically engineered *X. dendrorhous* strains for carotenoids production in high yields [32, 49, 56, 59, 61, 66]. Nevertheless, β-carotene and (3R, 3′R)-astaxanthin yields are still low. It is obviously necessary to generate more knowledge that addresses the limitations of the astaxanthin biosynthesis pathway [51, 62, 74].

Random mutagenesis using physical and chemical agents [8, 29, 66, 71, 78] have led to *X. dendrorhous* strains increase astaxanthin production [66]. Nevertheless, the targeted genes are usually unknown, which represents a disadvantage for the development of new metabolic engineering schemes to overproduce (3R, 3′R)-astaxanthin.

Genes involved in the carotenoids pathway have been induced to increase astaxanthin biosynthesis, but the results have not been enough. It is necessary to inactivate other genes such *CYP61* and/or *MIG1* (the latter involved in catabolic repression) and, in addition, induce protein domains such as *SRE1* N-terminal, indirectly related to the metabolism of carotenoids/astaxanthin [62, 95, 110, 111, 120, 121]. However, it is necessary to evaluate the multiple transformations directly on *X. dendrorhous* to increase the (3R, 3′R)-astaxanthin production. The improvement of astaxanthin overproduction is a request.

New metabolic engineering strategies on *X. dendrorhous* (such as those proposed in this review and others) could be implemented and studied to obtain more and new specific information of other regulatory mechanisms involved directly or indirectly to astaxanthin biosynthesis and, with this, achieve a genetic modification strategy capable of producing the compound in high yields.

## Data Availability

Not applicable.
